# Supplementary material for: Blood RNA biomarkers for tuberculosis screening in people living with HIV prior to anti-retroviral therapy initiation: A diagnostic accuracy study
Source: medRxiv. 2023 Jun 4:2023.06.01.23290783. Preprint. [Version 1] doi: 10.1101/2023.06.01.23290783 (PMC10312886; doi:10.1101/2023.06.01.23290783)
Supplement: Supplement 1 [file media-1.pdf]

Contents

Contents .... **Error! Bookmark not defined.**

Supplementary Table 1 3

Approach to calculation of blood RNA biomarker scores 3

Supplementary Table 2 4

Baseline characteristics of the study cohort, stratified by inclusion 4

Supplementary Table 3 6

Linear regression model showing factors associated with higher Suliman4 scores 6

Supplementary Table 4 7

Table showing thresholds where screening approaches have higher net benefit than confirmatory testing for all and none 7

Supplementary Table 5 8

Table showing greedy forward search looking for an optimised signature 8

Supplementary Figure 1 9

Sample size calculations 9

Supplementary Figure 2 10

Coefficients of variation for reference RNA samples, stratified by Nanostring codeset batch. 10

Supplementary Figure 3 11

Discrimination of TB signatures in BAR-TB dataset using Nanostring versus RNAseq (n=59). 11

Supplementary Figure 4 12

PCA of reference RNA samples 12

Supplementary Figure 5 13

Distribution of gene expression values normalized to GAPDH by Nanostring codeset batch 13

Supplementary Figure 6 14

Distribution of gene expression values normalized to GAPDH by Nanostring codeset batch after COMBAT correction 14

Supplementary Figure 7 15

Distribution of gene expression values normalized to GAPDH by Nanostring codeset batch after reference RNA normalization 15

Supplementary Figure 8 16

Discrimination of Roe3 and Zak11 signatures for (A) incipient and (B) presumptive TB using simple calculations (geometric means), compared to scaled and unscaled SVMs 16

Supplementary Figure 9 17

Interval time between enrolment to the study and first TB diagnosis or treatment initiation recorded in study or registry data. 17

Supplementary Figure 10 18

Linear regression model of predictors for Suliman4 scores 19

Supplementary Figure 11 21

Greedy forward search looking for an optimised signature 21

Supplementary Figure 12 22

Sensitivity analysis: Sputum culture or Ultra positivity 22

Supplementary Figure 13 23

Sensitivity analysis: Any positive TB test 23
Supplementary Figure 14..... 24
Sensitivity analysis: Recorded TB diagnosis or treatment within 6 months 24
Supplementary Figure 15..... 25
Sensitivity analysis: Using reference RNA-normalised data 25
References 26

Supplementary Table 1

Approach to calculation of blood RNA biomarker scores

All calculations are performed on log-2 transformed data. A single Nanostring probe was used to measure FCGR1A and FCGR1B.

Commented [GR1]: @Maddy - check wording
Commented [NM2R1]: fine

Signature	Calculation
BATF2[1]	BATF2
Gliddon3[2]	(FCGR1A + C1QB) - (ZNF296)
RISK6[3]	((GBP2 + FCGR1B + SERPING1)/3) - ((TUBGCP6 + TRMT2A + SDR39U1)/3))
Roe3[4]	(BATF2 + SCARF1 + GBP5)/3
Suliman4[5]	(GAS6 + SEPT4) - (CD1C + BLK)
Sweeney3[6]	(GBP5 + DUSP3)/2) - KLF2
Zak11[7]	Support vector machine model trained on original training dataset[8]

Supplementary Table 2

Baseline characteristics of the study cohort, stratified by inclusion

Characteristic	Overall, N = 862	Included, N = 707[1]	Excluded, N = 155[1]	p-value[2]
Age (years)	32 (26, 39)	32 (27, 39)	32 (25, 38)	0.2
Gender				0.6
Female	501 (58%)	407 (58%)	94 (61%)	
Male	360 (42%)	299 (42%)	61 (39%)	
Missing	1	1	0	
Previous TB	124 (14%)	98 (14%)	26 (17%)	0.4
CD4 (cells/mm3)	299 (172, 486)	306 (184, 486)	274 (129, 490)	0.12
Missing	8	5	3	
CD4 <200 cells/mm3	248 (29%)	193 (27%)	55 (36%)	0.041
Missing	8	5	3	
Haemoglobin (g/dl)	12.70 (11.30, 13.90)	12.70 (11.30, 13.90)	12.50 (11.25, 13.83)	0.4
Missing	177	150	27	
Body mass index (kg/m2)	24 (21, 29)	24 (21, 29)	23 (20, 29)	0.4
Missing	2	2	0	
Middle upper arm circumference (cm)	27.0 (25.0, 30.0)	27.0 (25.0, 30.0)	27.0 (25.0, 30.5)	0.7
WHO 4-symptom screen positive	487 (56%)	406 (57%)	81 (52%)	0.3
TBscoreII	1.00 (0.00, 2.00)	1.00 (0.00, 2.00)	1.00 (0.00, 1.25)	0.6
Missing	39	36	3	
CRP (mg/L)	6 (2, 32)	6 (2, 32)	6 (2, 34)	0.6
Missing	17	0	17	
Number of valid sputum cultures				0.7
0	37 (4.3%)	31 (4.4%)	6 (3.9%)	
1	50 (5.8%)	43 (6.1%)	7 (4.5%)	
2	775 (90%)	633 (90%)	142 (92%)	
Sputum culture positive	107 (13%)	89 (13%)	18 (12%)	0.8
Missing	37	31	6	
Sputum Ultra				0.4
Negative	754 (89%)	616 (88%)	138 (91%)	
Trace	19 (2.2%)	18 (2.6%)	1 (0.7%)	
Positive	78 (9.2%)	65 (9.3%)	13 (8.6%)	
Missing	11	8	3	
Urine LAM	24 (2.8%)	18 (2.6%)	6 (3.9%)	0.4
Missing	5	3	2	
Urine Ultra	45 (5.3%)	37 (5.3%)	8 (5.3%)	>0.9
Missing	10	6	4	
Sputum culture or Ultra positive	113 (13%)	94 (13%)	19 (12%)	0.9
Missing	12	9	3	
Any positive TB test	137 (16%)	112 (16%)	25 (16%)	>0.9
Missing	11	8	3	

Characteristic	Overall, N = 862	Included, N = 707[1]	Excluded, N = 155[1]	p-value[2]
Recorded TB diagnosis or treatment within 6 months	152 (18%)	130 (18%)	22 (14%)	0.3

[1]Statistics presented: median (IQR); n (%)

[2]Statistical tests performed: Wilcoxon rank-sum test; chi-square test of independence; Fisher's exact test

Supplementary Table 3

Linear regression model showing factors associated with higher Suliman4 scores

Characteristic	Beta	95% CI[1]	p-value
(Intercept)	3.8	2.2, 5.4	<0.001
Age (years)	0.01	0.00, 0.03	0.2
Gender			
Female	—	—	
Male	0.13	-0.23, 0.49	0.5
CD4 count (cells/mm3; per 10 unit increase)	-0.02	-0.03, -0.02	<0.001
Haemoglobin (g/dl)	-0.24	-0.32, -0.16	<0.001
Body mass index (kg/m2)	-0.01	-0.04, 0.01	0.4
Respiratory rate (per min)	0.07	0.02, 0.11	0.004
Number of W4SS symptoms	0.40	0.26, 0.55	<0.001
Sputum culture positive	1.5	1.0, 2.0	<0.001

[1]CI = Confidence Interval

Supplementary Table 4

Table showing thresholds where screening approaches have higher net benefit than confirmatory testing for all and none

Thresholds shown as threshold probabilities and as number willing to test ranges with confirmatory tests per true TB case detected.

Score	Threshold probability range	NWT range
CRP >= 5mg/L & Suliman4 >= Z2	0.04 - 0.29	3.5 - 23.8
Suliman4 >= Z2	0.04 - 0.23	4.3 - 23.8
CRP >= 5mg/L	0.04 - 0.2	5 - 22.2
W4SS	0.06 - 0.18	5.5 - 15.4

Supplementary Table 5

Table showing greedy forward search looking for an optimised signature

The full dataset was temporally split into 75%/25% development and validation sets. Increasing numbers of genes were iteratively added, in order of their discrimination for TB as single predictors.

Number of genes	Added gene	Logistic regression	Support vector machine	Disease risk score	Difference in geometric means
1	SEPT4	0.77 (0.66 - 0.88)	0.77 (0.66 - 0.88)	0.77 (0.66 - 0.88)	0.77 (0.66 - 0.88)
2	FCGR1A	0.77 (0.66 - 0.87)	0.73 (0.63 - 0.83)	0.77 (0.66 - 0.87)	0.77 (0.66 - 0.87)
3	SERPING1	0.76 (0.66 - 0.87)	0.75 (0.65 - 0.85)	0.76 (0.65 - 0.86)	0.76 (0.65 - 0.86)
4	BATF2	0.76 (0.65 - 0.86)	0.75 (0.64 - 0.85)	0.75 (0.65 - 0.86)	0.75 (0.65 - 0.86)
5	CD1C	0.75 (0.63 - 0.87)	0.5 (0.4 - 0.61)	0.76 (0.65 - 0.87)	0.75 (0.64 - 0.86)
6	DUSP3	0.76 (0.64 - 0.87)	0.71 (0.59 - 0.83)	0.76 (0.65 - 0.87)	0.75 (0.63 - 0.86)
7	C1QB	0.75 (0.63 - 0.86)	0.73 (0.62 - 0.84)	0.76 (0.65 - 0.86)	0.75 (0.63 - 0.86)
8	KLF2	0.76 (0.65 - 0.87)	0.71 (0.6 - 0.82)	0.76 (0.65 - 0.87)	0.76 (0.65 - 0.87)
9	ZNF296	0.78 (0.67 - 0.88)	0.77 (0.67 - 0.87)	0.76 (0.65 - 0.87)	0.77 (0.66 - 0.88)
10	GBP1	0.78 (0.68 - 0.88)	0.75 (0.64 - 0.85)	0.76 (0.65 - 0.87)	0.77 (0.66 - 0.88)
11	GAS6	0.77 (0.67 - 0.88)	0.74 (0.63 - 0.86)	0.77 (0.66 - 0.88)	0.77 (0.66 - 0.88)
12	GBP5	0.8 (0.71 - 0.89)	0.76 (0.66 - 0.87)	0.77 (0.66 - 0.87)	0.77 (0.66 - 0.88)
13	SDR39U1	0.8 (0.71 - 0.89)	0.76 (0.66 - 0.86)	0.77 (0.66 - 0.88)	0.77 (0.66 - 0.89)
14	SCARF1	0.78 (0.69 - 0.88)	0.74 (0.64 - 0.85)	0.76 (0.65 - 0.87)	0.77 (0.66 - 0.88)
15	TRMT2A	0.79 (0.69 - 0.88)	0.74 (0.64 - 0.85)	0.77 (0.66 - 0.88)	0.77 (0.66 - 0.88)
16	ETV7	0.78 (0.68 - 0.88)	0.74 (0.63 - 0.85)	0.76 (0.65 - 0.87)	0.77 (0.66 - 0.88)
17	TAP1	0.78 (0.68 - 0.88)	0.74 (0.64 - 0.85)	0.76 (0.65 - 0.87)	0.77 (0.66 - 0.88)
18	GBP2	0.78 (0.68 - 0.88)	0.74 (0.63 - 0.84)	0.76 (0.65 - 0.87)	0.77 (0.66 - 0.88)
19	BLK	0.78 (0.68 - 0.87)	0.74 (0.64 - 0.85)	0.76 (0.65 - 0.87)	0.76 (0.65 - 0.87)
20	TUBGCP6	0.78 (0.68 - 0.87)	0.75 (0.64 - 0.85)	0.76 (0.65 - 0.87)	0.77 (0.66 - 0.88)
21	STAT1	0.77 (0.68 - 0.87)	0.74 (0.63 - 0.85)	0.76 (0.65 - 0.87)	0.77 (0.66 - 0.88)
22	TRAFD1	0.78 (0.69 - 0.88)	0.76 (0.66 - 0.86)	0.76 (0.65 - 0.87)	0.76 (0.65 - 0.87)

Supplementary Figure 1

Sample size calculations

Relationship between total sample size required and proportion of positive cases within the sample stratified by target area under the receiver operating characteristic curve (AUROC) to discriminate between cases and controls with 95% lower bound confidence interval of 0.05 from target AUROC. Dashed line represents sample size of present study (N=707).

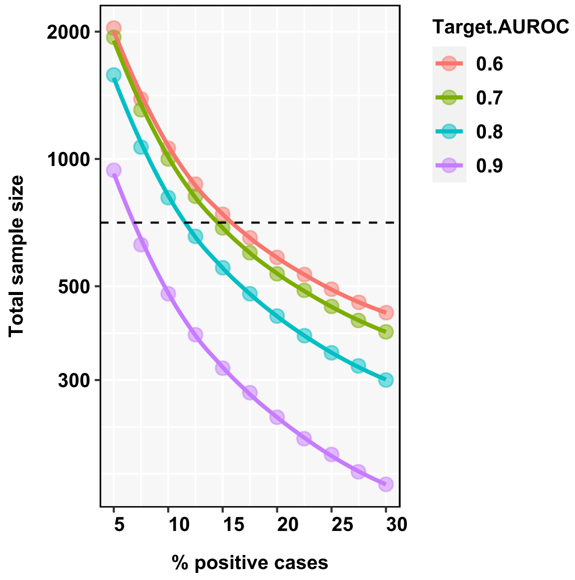

Supplementary Figure 2

Coefficients of variation for reference RNA samples, stratified by Nanostring codeset batch.

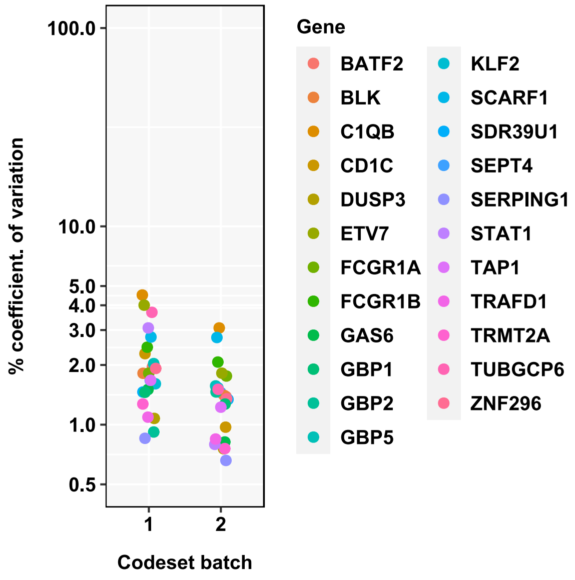

Supplementary Figure 3

Discrimination of TB signatures in BAR-TB dataset using Nanostring versus RNAseq (n=59).

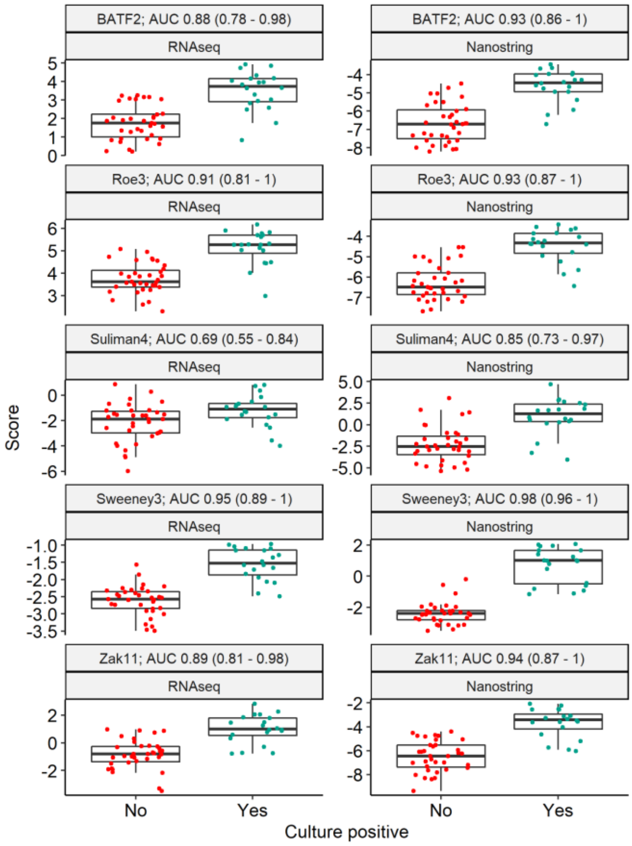

Supplementary Figure 4

PCA of reference RNA samples

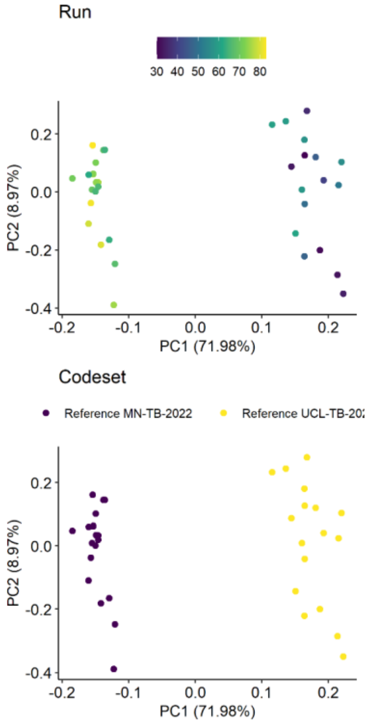

Supplementary Figure 5

Distribution of gene expression values normalized to GAPDH by Nanostring codeset batch

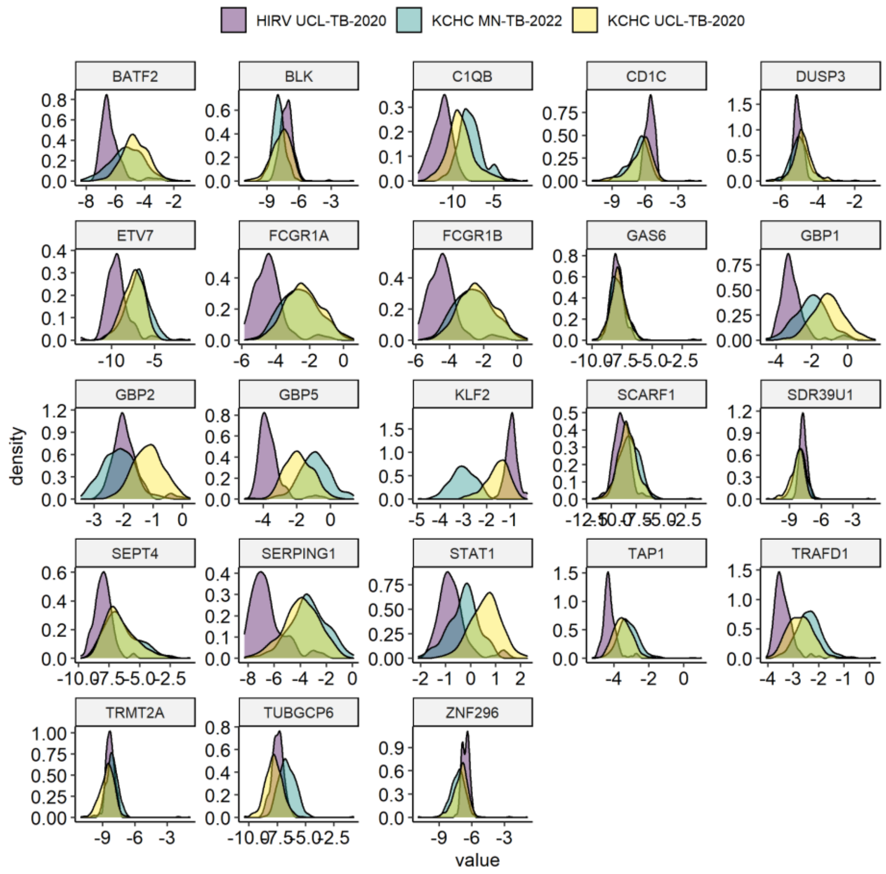

Supplementary Figure 6

Distribution of gene expression values normalized to GAPDH by Nanostring codeset batch after COMBAT correction

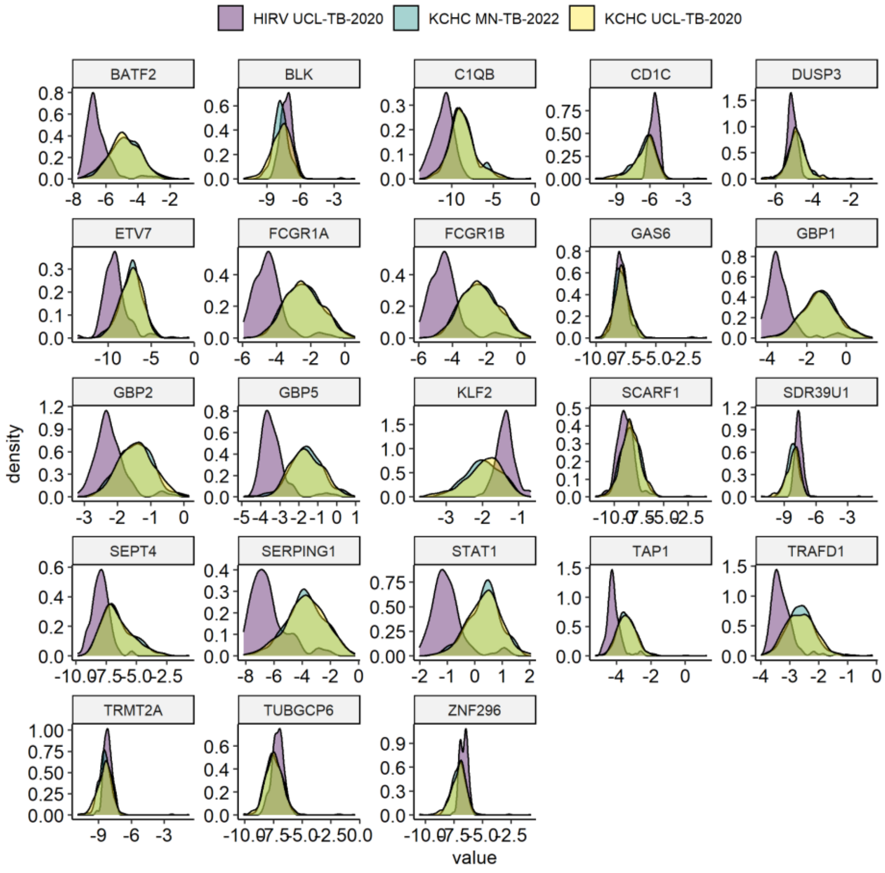

Supplementary Figure 7

Distribution of gene expression values normalized to GAPDH by Nanostring codeset batch after reference RNA normalization

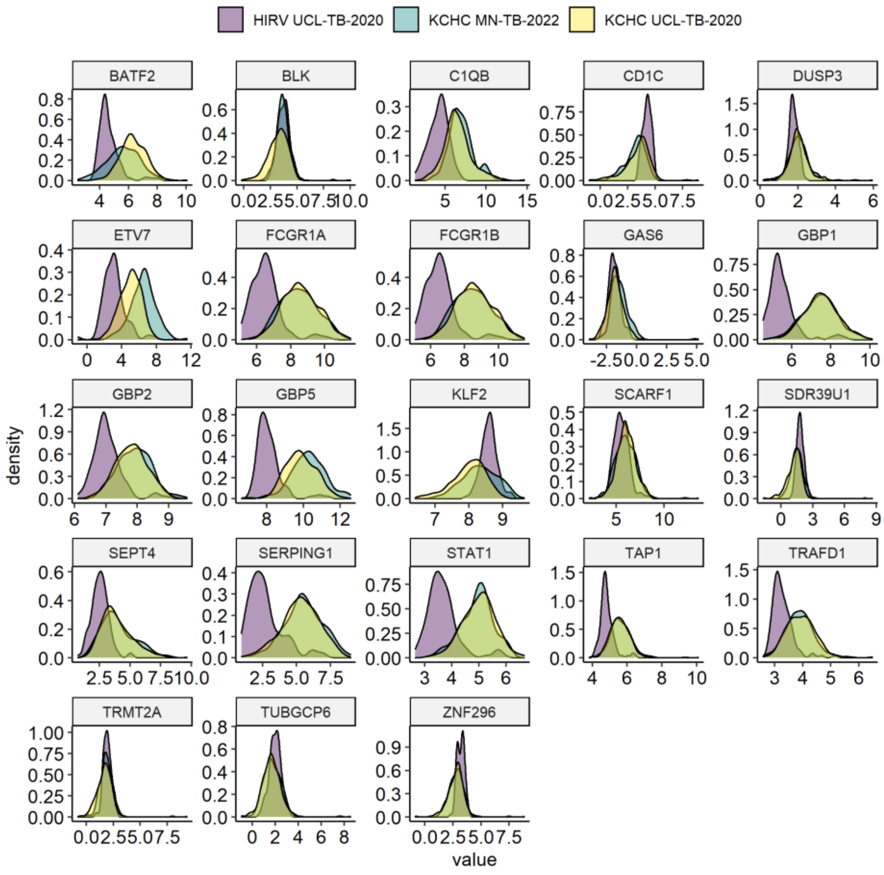

Supplementary Figure 8

Discrimination of Roe3 and Zak11 signatures for (A) incipient and (B) presumptive TB using simple calculations (geometric means), compared to scaled and unscaled SVMs

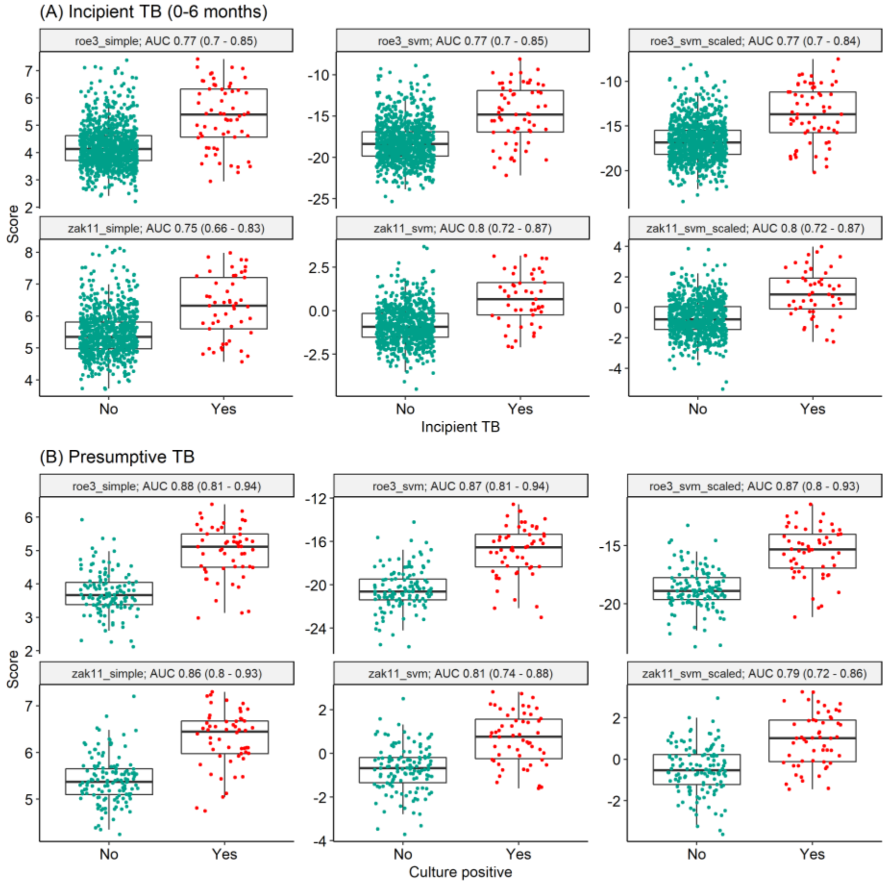

Supplementary Figure 9

Interval time between enrolment to the study and first TB diagnosis or treatment initiation recorded in study or registry data.

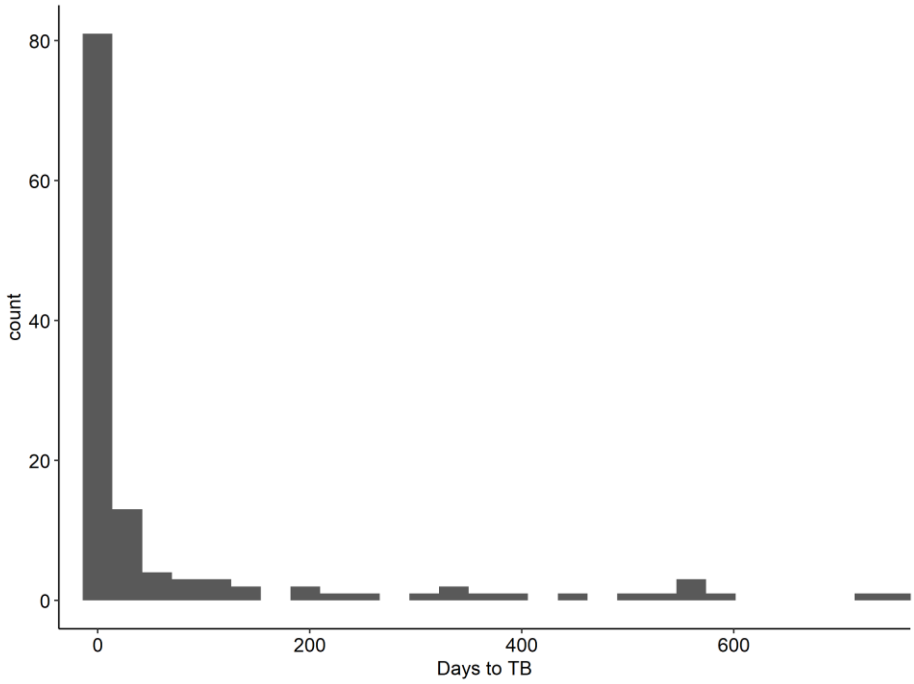

Supplementary Figure 10

Co-correlation of blood RNA biomarkers and CRP

Spearman rank co-correlation matrix of blood RNA biomarkers and CRP (n=707), with hierarchical clustering using complete linkage method.

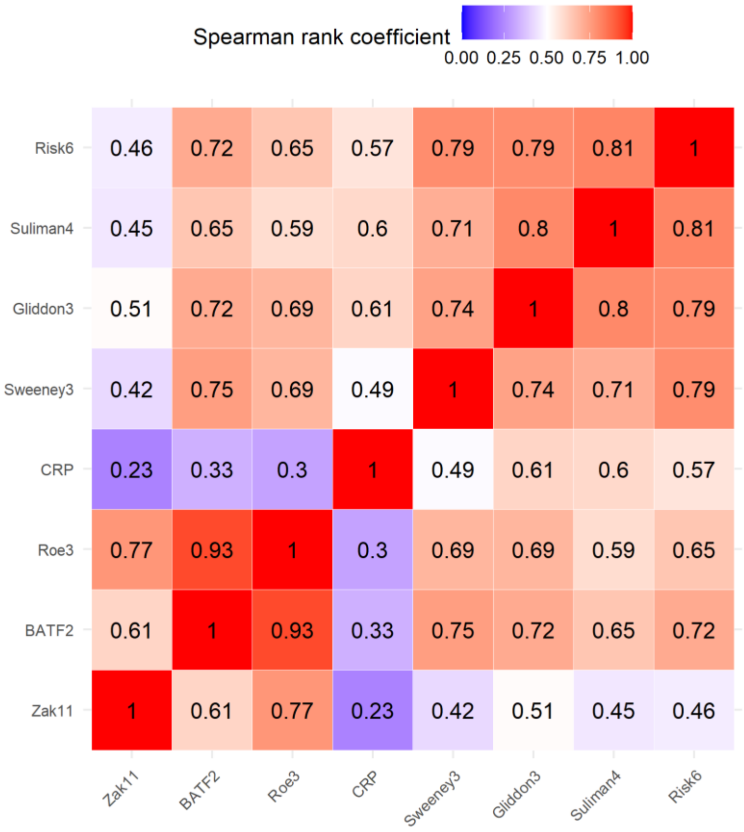

Supplementary Figure 11

Associations between Suliman4 blood RNA biomarker Z-score sand indices of HIV/TB disease severity

Individual scatter plots of Suliman4 blood RNA biomarker Z score with selected variables (indicated) associated with HIV and/or TB disease burden/severity, for all participant data (N=676) stratified by sputum Mtb culture result. Spearman correlation coefficients and p values are shown for each pairwise analysis. Sputum smear grade is shown as 1 (scanty), 2 (+), 3 (++) and 4 (+++)..

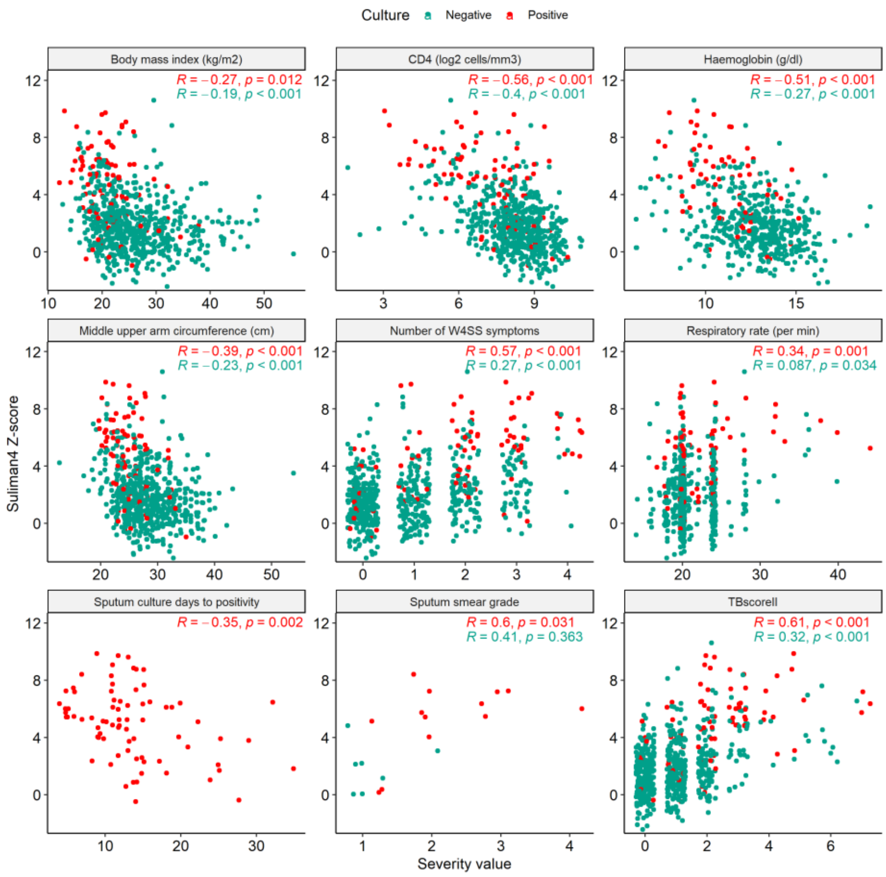

Supplementary Figure 12

Linear regression model of predictors for Suliman4 scores

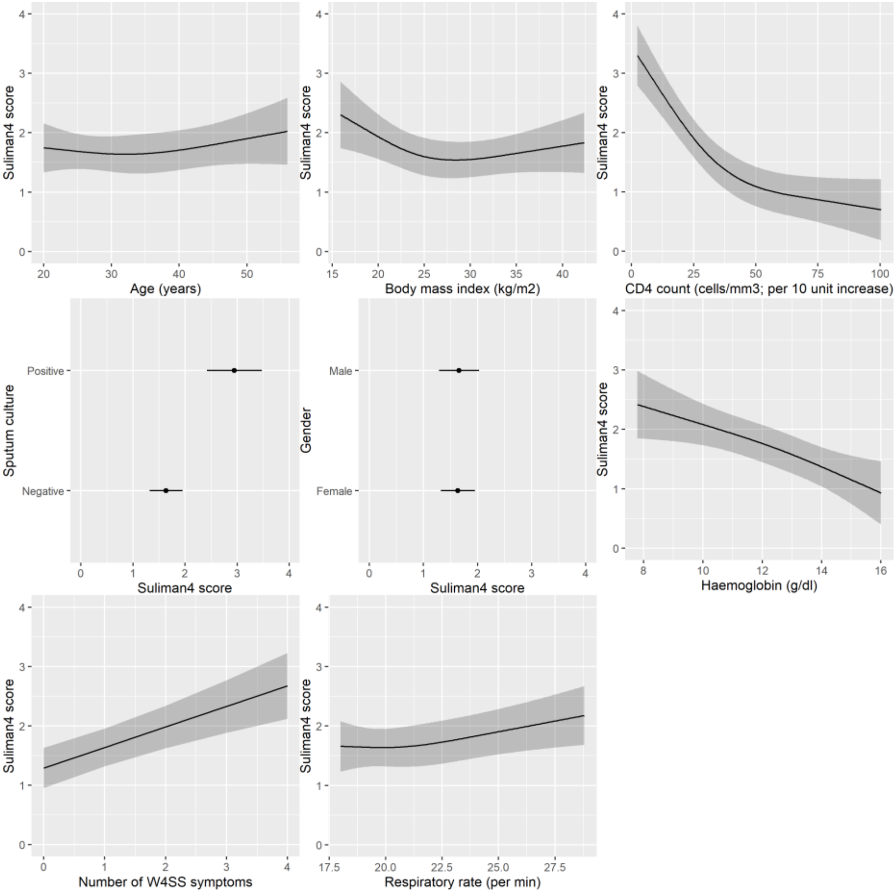

Supplementary Figure 13

Greedy forward search looking for an optimised signature

The full dataset was temporally split into 75%/25% development and validation sets. Increasing numbers of genes were iteratively added, in order of their discrimination for TB as single predictors.

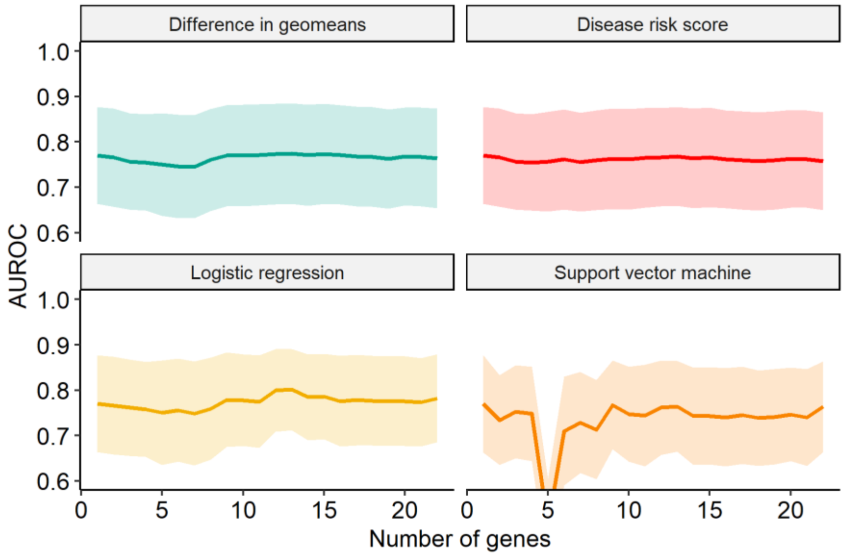

Supplementary Figure 14

Sensitivity analysis: Sputum culture or Ultra positivity

Scores and discrimination of RNA signatures for secondary outcome of sputum culture or Ultra positivity (n = 698 participants). Scores are shown as Z-scores for RNA signatures, and log-2 transformed CRP (mg/L). Discrimination presented as area under the receiver operating characteristic curve (AUC), with 95% confidence intervals.

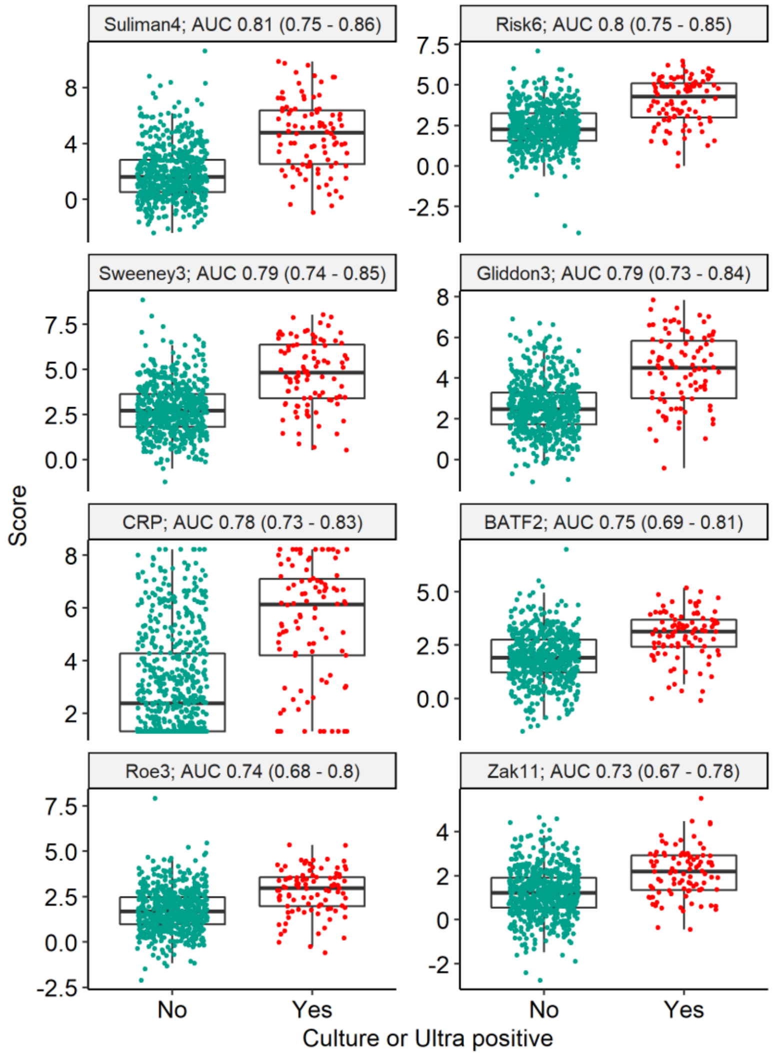

Supplementary Figure 15

Sensitivity analysis: Any positive TB test

Scores and discrimination of RNA signatures for secondary outcome of any positive TB test, including urine LAM and urine Ultra (n = 699 participants). Scores are shown as Z-scores for RNA signatures, and log-2 transformed CRP (mg/L). Discrimination presented as area under the receiver operating characteristic curve (AUC), with 95% confidence intervals.

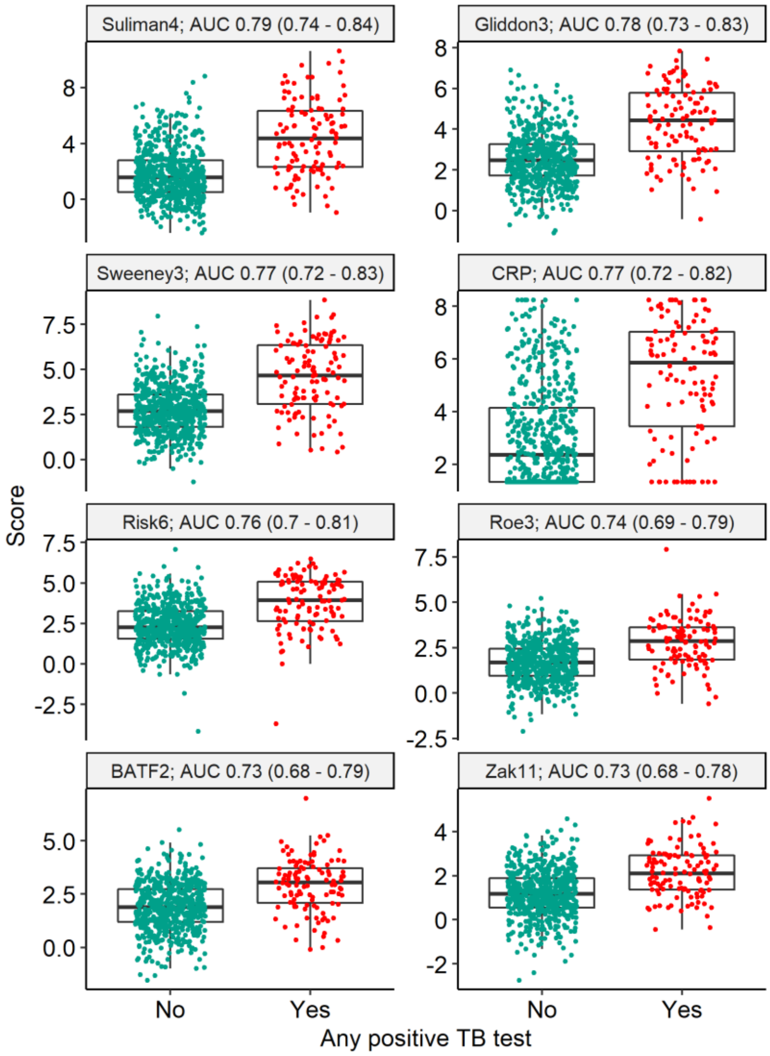

Supplementary Figure 16

Sensitivity analysis: Recorded TB diagnosis or treatment within 6 months

Scores and discrimination of RNA signatures for secondary outcome of recorded TB diagnosis or treatment within 6 months (n = 707 participants). Scores are shown as Z-scores for RNA signatures, and log-2 transformed CRP (mg/L). Discrimination presented as area under the receiver operating characteristic curve (AUC), with 95% confidence intervals.

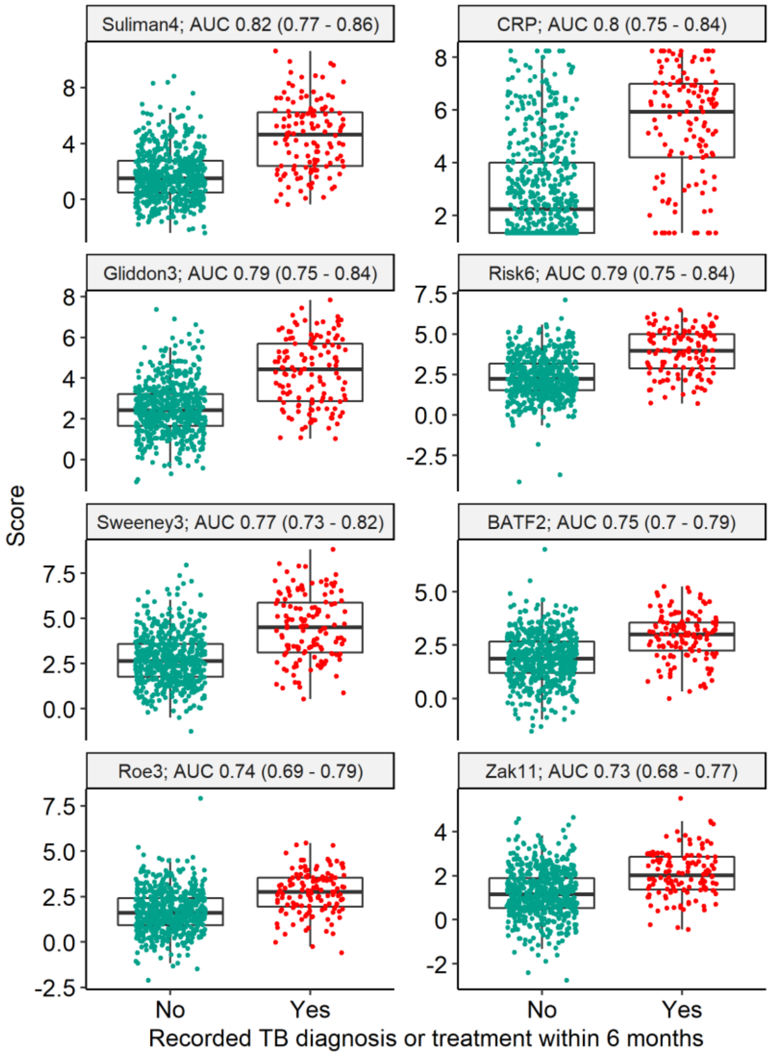

Supplementary Figure 17

Sensitivity analysis: Using reference RNA-normalised data

Scores and discrimination of RNA signatures for primary outcome of sputum culture (n = 676 participants), using reference RNA normalisation of Nanostring data as sensitivity analysis. Scores are shown as untransformed scores for RNA signatures, and log-2 transformed CRP (mg/L). Discrimination presented as area under the receiver operating characteristic curve (AUC), with 95% confidence intervals.

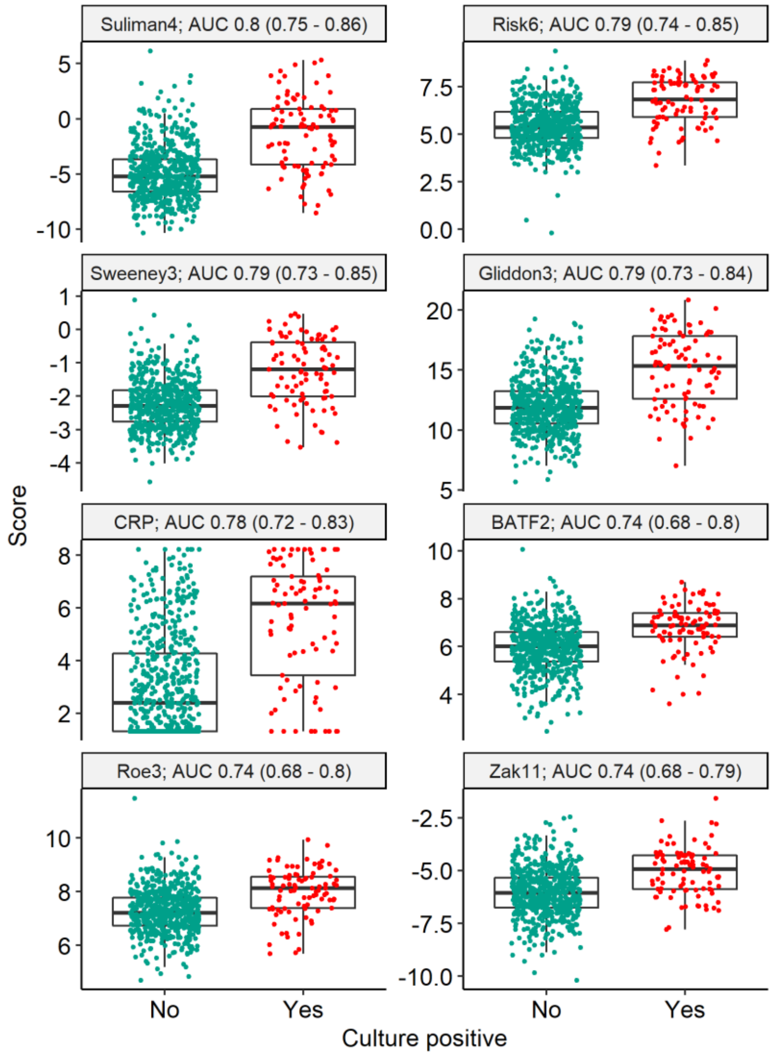

References

1 Roe JK, Thomas N, Gil E, *et al.* Blood transcriptomic diagnosis of pulmonary and extrapulmonary tuberculosis. *JCI insight* 2016; **1**: e87238.

2 Gliddon HD, Kaforou M, Alikian M, *et al.* Identification of reduced host transcriptomic signatures for tuberculosis and digital PCR-based validation and quantification. *bioRxiv* 2019; : 583674.

3 Penn-Nicholson A, Mbandi SK, Thompson E, *et al.* RISK6, a 6-gene transcriptomic signature of TB disease risk, diagnosis and treatment response. *Sci Rep* 2020; **10**: 1–21.

4 Roe J, Venturini C, Gupta RK, *et al.* Blood transcriptomic stratification of short-term risk in contacts of tuberculosis. *Clin Infect Dis* 2019; published online March 28. DOI:10.1093/cid/ciz252.

5 Suliman S, Thompson E, Sutherland J, *et al.* Four-gene Pan-African Blood Signature Predicts Progression to Tuberculosis. *Am J Respir Crit Care Med* 2018; **197**: 1198–208.

6 Sweeney TE, Braviak L, Tato CM, Khatri P. Genome-wide expression for diagnosis of pulmonary tuberculosis: a multicohort analysis. *Lancet Respir Med* 2016; **4**: 213–24.

7 Darboe F, Mbandi SK, Thompson EG, *et al.* Diagnostic performance of an optimized transcriptomic signature of risk of tuberculosis in cryopreserved peripheral blood mononuclear cells. *Tuberculosis* 2018; **108**: 124–6.

8 Zak DE, Penn-Nicholson A, Scriba TJ, *et al.* A blood RNA signature for tuberculosis disease risk: a prospective cohort study. *Lancet* 2016; **387**: 2312–22.
